# Dendritic Cells in Tolerance and Immunity against Pathogens

**DOI:** 10.1155/2016/6438036

**Published:** 2016-03-31

**Authors:** Silvia Beatriz Boscardin, Daniela Santoro Rosa, Alice O. Kamphorst, Christine Trumpfheller

**Affiliations:** ^1^Laboratory of Antigen Targeting to Dendritic Cells, Department of Parasitology, Institute of Biomedical Sciences, University of São Paulo, 05508-000 São Paulo, SP, Brazil; ^2^Department of Microbiology, Immunology and Parasitology, Federal University of São Paulo (UNIFESP/EPM), 04023-062 São Paulo, SP, Brazil; ^3^Emory Vaccine Center and Department of Microbiology and Immunology, Emory University School of Medicine, 1510 Clifton Road Rm G214, Atlanta, GA 30322, USA; ^4^Laboratory of Cellular Physiology and Immunology and Chris Browne Center, The Rockefeller University, 1230 York Avenue, New York, NY 10065, USA

Dendritic cells (DCs) are a highly specialized population of antigen-presenting cells that play a key role in the induction of adaptive immune responses against different pathogens and in the maintenance of peripheral tolerance [1, 2]. Since their discovery by Ralph Steinman in the 70s [3–6], much has been learned about their ontogeny, migration, and antigen presentation capacities in lymphoid and nonlymphoid organs [7]. Advances in flow cytometry allowed the selection of surface markers that help distinguish different DC subsets [8]. In addition, the identification of many receptors has added information on how these cells sense the environment and respond to different stimuli [9]. The role of DCs in the induction of immune responses and/or in peripheral tolerance has been extensively studied, particularly in mice, using different models that include infectious diseases and cancer, but also autoimmune diseases. In addition, progress has been made in our understanding of human DC subsets.

In this special issue, a number of review articles will illustrate our current understanding of how DCs are affected in different contexts, ranging from inflammatory to more tolerogenic settings. For example, J. M. Motta and V. M. Rumjanek will discuss how different environments affect DC function. They focus especially on how DC function is modulated in the presence of tumors (a tolerogenic setting) or in the presence of organ transplantation (a more proinflammatory setting). The role of DCs following heart transplantation is also revised by M.-T. Dieterlen et al., when they additionally discuss in detail DC function in hypertension, atherosclerosis, and heart failure. On the other hand, S. Winning and J. Fandrey discuss how hypoxia modulates DC function. This is an important topic because antigen presentation normally takes place in organs and tissues that exhibit low oxygen tension, and we are just starting to recognize hypoxia as a key factor on the modulation of immune responses. On a more tolerant setting, we will learn from A. Steimle and J.-S. Frick how intestinal DCs interact constantly with different species of commensal bacteria and how these bacteria can regulate DC phenotype. DC function and tolerance break in systemic lupus erythematosus are addressed by X. Liao et al. Recent evidence suggests that DC activation by self-antigens contributes to tolerance breakdown and to the induction of lupus pathogenesis. The role of DCs in different contexts of infection is also the focus of three reviews in this special issue. N. A. Mabbott and B. M. Bradford address how prions may exploit conventional DCs to infect the host, while D. Feijó et al. and K. N. S. Amorim et al. focus on DC interactions with two different intracellular parasites. Interactions between DCs and parasites from the genus* Leishmania* are discussed by D. Feijó et al., while K. N. S. Amorim et al. overview the importance of DCs during* Plasmodium* infection and how they sense different parasite components. Finally, I. G. Zizzari et al. specifically address how a DC receptor recognizes modified glycoproteins expressed by tumors and highlight the importance of antigen structure in the modulation of DC mediated immune responses.

New information is also provided by this special issue. T. Bertran et al. analyze interactions between* Gardnerella vaginalis* and human monocyte-derived DCs, while D. Clarke et al. study how DCs in contact with group B* Streptococcus* modulate activation of CD4^+^ T cells.

In conclusion, this special issue highlights different functions of DCs in complex scenarios such as immunity and tolerance. Understanding how DCs help our immune system to deal with infections and to maintain the steady state is important, and such knowledge may be used in the design of better vaccines and in the treatment of autoimmune diseases.

*Silvia Beatriz Boscardin* *Silvia Beatriz Boscardin* *Daniela Santoro Rosa* *Daniela Santoro Rosa* *Alice O. Kamphorst* *Alice O. Kamphorst* *Christine Trumpfheller* *Christine Trumpfheller*
